# Polypharmacy in the oldest old (≥80 years of age) patients in China: a cross-sectional study

**DOI:** 10.1186/s12877-018-0754-y

**Published:** 2018-03-02

**Authors:** Xiaoxing Lai, Hongwei Zhu, Xiaopeng Huo, Zheng Li

**Affiliations:** 1 Department of Health Care, Peking Union Medical College Hospital, Beijing, 100730 China; 2 School of Nursing, Peking Union Medical College, Beijing, 100730 China

**Keywords:** Oldest old, Polypharmacy, Non-adherence, Medication knowledge

## Abstract

**Background:**

The oldest old generally have worse health and more comorbidities than the general population of older adults, and they are more likely to be exposed to polypharmacy. Reliable investigation of polypharmacy among the oldest old (≥80 years of age) in China are lacking. So this study aims to describe the polypharmacy status of oldest old patients ≥80 years of age and to assess the factors influencing medication compliance.

**Methods:**

This was a cross-sectional study of 258 oldest old patients ≥ 80 years of age and hospitalized at a tertiary hospital in Beijing between December 1, 2014 and June 30, 2015. They completed three validated questionnaires to assess their pre-admission status (general demographics, medication knowledge, and medication adherence). Potentially inappropriate medications (PIM) use was evaluated by physicians.

**Results:**

The majority of the patients (55.4%) took < 10 types of drugs. The numbers of drugs taken ranged from 8 to 60 drugs (median of 22.9). Patients taking 11–20 drugs accounted for 46.1% of the patients. Subjects with a history of adverse drug reactions accounted for 40.3%. The proportion of PIMs was 27.1%. Compliance was only 32.6% among the oldest old patients with polypharmacy. Age and medication classes were independently negatively associated with compliance, and medication knowledge was independently positively associated with compliance.

**Conclusion:**

Oldest old patients (≥ 80 years of age) had a poor medication knowledge. Age, medication classes, and medication knowledge were independently associated with medication compliance.

**Electronic supplementary material:**

The online version of this article (10.1186/s12877-018-0754-y) contains supplementary material, which is available to authorized users.

## Background

The China Report of the Development on Aging Cause Blue Book [1] reported that the number of old individuals in China exceeded 200 million in 2013, and that up to 22.73 million of these individuals were ≥80 years old. The Aging Committee of China predicts that the number of oldest old ≥80 years old in China will reach 30.67 million by 2020, accounting for 12.37% of the old population.

Harboring multiple comorbidities is a common fact among the older adults, who often have to be treated with a combination of drugs, leading to polypharmacy. Currently, there is no universally accepted definition of polypharmacy, although it is generally considered as being the use of ≥5 types of medications [1–3]. In the nursing home setting, an American study showed that 34.8% of the patients > 85 years of age were taking at least nine drugs [4]. A Canadian study showed that 15.5% of the nursing home residents were on at least nine medications [5]. In China, a study showed that among patients of 80–104 years of age, the medication exposure rate was 100%, with 70% of them taking at least 6 drugs [6].

Despite the fact that drugs are prescribed to deal with specific diseases and comorbidities, polypharmacy is associated with consequences detrimental to the patient and with a burden to the society. A study showed that polypharmacy was associated with an increase of 30% in medical costs [7]. In addition, taking multiple drugs increases the risk of adverse reactions and drug interactions. In 2005, it has been estimated that 4.3 million medical consultations were directly associated with drug adverse reactions [8], with 10% of all emergency admission being due to an adverse reaction [9]. About 40% of hospitalized older adults will experience an adverse drug reaction [8]. A study showed that older adults taking 5–9 drugs had a 50% probability of drug interaction, increasing to 100% for those taking ≥ 20 drugs [10]. Among older adults, polypharmacy has been associated with declining functional status, cognitive impairment, falls, urinary incontinence, and impaired nutritional status [11–13]. These adverse outcomes of polypharmacy have also been observed in Chinese oldest old ≥ 80 years of age [6].

Non-adherence is one of the most important negative impacts of polypharmacy, with rates of 43–100% among different communities of oldest old patients [14, 15]. Non-adherence may have dire impacts on the patients’ life [14, 16, 17]. Ulfvarson et al. [18] claimed that only 30% of Swedish older adults who had been discharged for one week fully adhered to medical instructions regarding their prescribed drugs. Therapeutic failure leading to hospitalization, additional drugs, additional medical/surgical procedures, and increased health costs (both to the patient and society) are important consequences of treatment non-adherence [14, 16, 17].

Reliable knowledge of polypharmacy among the oldest old (≥80 years of age) in China is lacking. The oldest old generally have worse health and more comorbidities than the general population of older adults, and they are more likely to be exposed to polypharmacy [4, 5, 19]. In addition, a better understanding of what influences older adults’ level of medication compliance may help health care providers to find appropriate ways to improve medication compliance. This question is particularly important because of the aging population in China and in the world in general. Therefore, the aims of the present study were to describe the polypharmacy status of the oldest old patients (≥80 years of age) and to assess the factors influencing medication compliance in patients with polypharmacy in Beijing.

## Methods

### Study design and subjects

This was a cross-sectional study (convenience sample) of 258 oldest old patients (≥ 80 years of age) and hospitalized at a tertiary hospital in Beijing between December 1, 2014 and June 30, 2015. Their pre-admission status was assessed using three validated questionnaires. The study was approved by the ethics committee of the hospital. All patients provided a written informed consent.

The inclusion criteria were: 1) ≥80 years of age; 2) at least one chronic disease, selected basing on “China Report of the Development on Aging Cause Blue Book” [1] and “Report of Status and Trend in Development of Chronic Diseases in Older Adults in China”, which was published by China Research Center on Aging (hypertension, coronary heart disease, diabetes mellitus, hyperlipidemia, chronic gastritis, chronic low back pain, atherosclerosis, respiratory disease, or skin or tissue disease); and 3) taking ≥5 types of medications before admission (including prescription medications but not including non-prescription medications or over-the-counter health care products). The exclusion criteria were: 1) life-threatening or terminal disease; 2) mental disorder or cognitive impairment; 3) or unwilling to participate.

### Questionnaires and data collection

Following an explanation of the purpose of the study, consent was obtained from patients who met the eligibility criteria and agreed to participate. Demographic and medical data were obtained from the patients and their medical records. The three questionnaires took approximately 20 min to fill and the patients filled the questionnaires at the hospital. Pill boxes were provided as an incentive for participation. Before the patients completed the questionnaires, the meaning and requirement of all items were explained in details by a healthcare professional. The questionnaires were filled by the patients, without help, and in the presence of the professional. All questionnaires covered the pre-admission period.

The general demographic questionnaire included sex, age, educational level, marital status, and payment of medical expenses. The subjects also provided a self-evaluation of their health status, the types and number of diseases they had, and the number and names of the drugs they were taking. This information was validated using the patient’s medical records. For analysis convenience, medications were recorded and coded according to “Practical Drug Code for Hospital Pharmacy” which is occupation standard of medicine in China and used in hundreds of medical organizations, compiled and published by Pharmacy Department of Peking Union Medical College Hospital.. History of adverse reactions (ADR) was obtained from the patients’ medical records and the ADR Monitoring System of the hospital. ADR monitoring system was based on Hospital Information System (HIS) System of internal network in Peking Union Medical College Hospital. Electronic system simplified the procedure of reporting and recorded all the ADRs roundly. The reporter could inquire about the content of the report and consult the feedback content in time by the system.

The medication knowledge questionnaire was developed by the authors based on an extensive literature review of Chinese and English papers [20–27], relevant work experience, and suggestions from five geriatrists and pharmacists. The resulting questionnaire was revised by a group of faculty members from a hospital in Beijing. The content validity was evaluated by five geriatrists and pharmacists. A strong positive correlation was defined as 4 points, positive correlation as 3 points, weak correlation as 2 points, and no correlation as 1 points. The content validity index (CVI) of the questionnaire was 0.96, and the CVI of the items ranged from 0.71–1.00. The Cronbach’s α was calculated as a measure of the internal validity of the questionnaire and was 0.82. The questionnaire (English translation, non-validated) is presented as Additional file 1. The questionnaire was also assessed for ease of comprehension and readability by 15 oldest old patients with polypharmacy (not participating in the present study).

This questionnaire included eight dimensions and 25 questions. The content of the items addressed the subjects’ knowledge about: 1) medication delivery modes; 2) medication dosage; 3) the importance of taking medications regularly; 4) drug side effects; 5) the dangers of drug abuse (self-medication without a doctor’s guidance and/or beyond the medical scope of treatment and dose standards); 6) how to identify expired drugs; 7) drug incompatibilities; and 8) drug storage. The average awareness level was calculated as the number of questions answered correctly divided by the total number of questions.

The 4-item Morisky scale was used to assess medication compliance [28]. Xu et al. [29] evaluated the internal reliability of the Chinese version of the Morisky scale in Chinese patients and reported a Cronbach’s α of 0.715. The scale consists of four questions regarding the 6 months prior to admission: 1) Have you ever forgotten to take your medicine? 2) Are you careless about taking your medicine? 3) Do you sometimes stop taking your medicine when you feel better? 4) Sometimes, if you feel worse when you take your medicine, do you stop taking it? The patients were considered adherent if their answers to all four questions were “No”, and they were considered poorly adherent if one or more of their responses was “Yes”. The rate of medication adherence was defined as the number of compliant subjects divided by the total number of subjects.

### Potentially inappropriate medications (PIMs)

The 2012 American Geriatrics Society’s updated Beers criteria [30] were used to assess PIMs. The new 2012 Beers criteria were translated into Chinese and adapted in accordance with the National Essential Drugs List of China (2009 edition), omitting medicines unapproved in China [31]. PIMs were defined as drugs with a potential for harm that outweighed their benefits. The medication review chart was used to assess PIMs comprehensively, and the medication review chart (English translation, non-validated) is presented as Additional file 2. In this study, PIM was evaluated by five attending physicians (all had medical doctor degree, and worked in geriatric wards as attending physicians at least 5 years). At least one inappropriate medication in the patient’s prescription regimen, as based on the 2012 Beers criteria, was considered to indicate the use of PIMs.

### Data analysis

Data were analyzed with descriptive and inferential statistics using SPSS 17.0 (IBM, Armonk, NY, USA). Descriptive statistics were reported as mean, standard deviation, proportion, and percentage. Inferential procedures were also used including univariate and multivariate logistic regression. Two-sided *P*-values < 0.05 were considered statistically significant.

## Results

### Characteristics of the subjects

Of the 258 subjects, the age range was from 80~ 109; mean age was 88.7 ± 6.5 years. Among the subjects, males represented 58.9%. The majority (55.8%) of the patients lived with their spouses and 96.9% of the subjects received free medical care or were covered by health insurance. The majority of patients (53.9%) had junior college diplomas, and 24.7% had middle-school education or below (Table 1).

**Table 1:** Baseline characteristics of oldest old patients > 80 years of age with polypharmacy (*n* = 258)

Index	Stratification	Mean ± SD	*n*	%
Age (years)		88.7 ± 6.5		
Gender	Male		152	58.9
	Female		106	41.1
Marital status	Have spouse		161	62.4
	No spouse		97	37.6
Living status	Living alone		16	6.2
	Living with spouse		144	55.8
	Living with housekeeper		42	16.3
	Living with offspring		56	21.7
Medical expenses	Self-paid		8	3.1
	Health insurance		99	38.4
	Publicly paid		151	58.5
Health condition	Good		29	11.2
	Moderate		111	43.0
	Poor		118	45.7
Number of diagnosed diseases		7.0 ± 2.3		

### Chronic diseases

The number of chronic diseases was 3–13/subject (mean of 7.0 ± 2.3). The five most common chronic diseases were hypertension (*n* = 160, 62.0%), hyperlipidemia (*n* = 112, 43.4%), atherosclerosis (*n* = 111, 43.0%), chronic gastritis (*n* = 106, 41.4%), and coronary heart disease (*n* = 105, 40.7%) (Table 2).

**Table 2:** Distribution of chronic diseases

Rank	Chronic disease	*n*	%
1	Hypertension	160	62.0
2	Hyperlipidemia	112	43.4
3	Atherosclerosis	111	43.0
4	Chronic gastritis	106	41.1
5	Coronary heart disease	105	40.7
6	Diabetes mellitus	98	38.0
7	Skin and tissue disease	87	33.7
8	Chronic low back pain	75	29.1
9	Respiratory diseases	69	26.7

### Medications

The five most commonly used prescription drugs were: gastrointestinal drugs (*n* = 199), vitamins and minerals (*n* = 185), traditional Chinese medications (TCM, refer particularly to Chinese patent medicines, which were produced by traditional Chinese herbal medicine according to the formulary prescription and preparation process, and had definite and systemic effects.) (*n* = 156), osteoporosis prevention drugs (*n* = 143), and antithrombotic drugs (*n* = 142) (Table 3).

**Table 3:** Distribution of drugs regularly taken by the patients

Rank	Drug	*n*	%
1	Gastrointestinal drugs	199	77.1
2	Vitamins and minerals	185	71.7
3	Traditional Chinese medicine	156	60.5
4	Osteoporosis preventing drugs	143	55.4
5	Antithrombotic drugs	142	55.0
6	β-blockers	120	46.5
8	Anti-constipation drugs	102	39.5
9	Prostate treatment drugs	98	38.0
10	Statins	69	26.7

The maximum number of types of medications used by the oldest old with polypharmacy was 27. The majority of the subjects (55.4%) took < 10 types of drugs. Patients taking 11–20 pills/tablets of drugs accounted for 46.1% of the patients. Subjects with a history of adverse drug reactions accounted for 40.3%. In addition, 60.5% of the subjects took Chinese traditional medicines, and 64.7% of the subjects used over-the-counter health care products (Table 4).

**Table 4:** Number of drug types, drug numbers, frequencies of adverse drug reactions, and use of Chinese traditional medicines and health care products

Index	Stratification	*n*	%
Drug types	< 10	143	55.4
	10–15	59	22.9
	> 15	56	21.7
Number of drug types	≤10	9	3.5
	11–20	119	46.1
	21–30	81	31.4
	> 30	49	19.0
Adverse drug reactions	Yes	104	40.3
	No	95	36.8
	Uncertain	59	22.9
Use of Chinese traditional medicines	Yes	156	60.5
	No	102	39.5
Use of over-the-counter health care products	Yes	167	64.7
	No	91	35.3

### Medication knowledge

The average accuracy of the subjects’ responses to the medication knowledge items was 68.7%. The items with the highest average numbers of correct responses included those on expired drugs, medication types, and drug storage, whereas the items with the lowest awareness were about drug side effects, drug abuse, drug incompatibility, and medication dosage (Table 5). Correlations were identified between age and medication knowledge; medication types and medication knowledge; and numbers of drugs and medication knowledge. Medication knowledge declined with increasing age: for each one-unit increase in age, the average medication knowledge score decreased by 0.697 (*r* = − 0.697, *P* < 0.05).

**Table 5:** Medication knowledge among oldest old patients > 80 years of age with polypharmacy

Dimension (points)	Score	Average awareness rate (%)
Total score (100)	68.7 ± 12.2	68.7
Medication mode (8)	5.7 ± 2.2	71.3
Medication dosage (12)	8.1 ± 3.4	67.5
Regular medication (12)	8.5 ± 2.8	70.8
Drug side effects (12)	6.7 ± 2.8	55.8
Drug abuse (16)	10.1 ± 3.5	63.1
Expired drugs (16)	12.3 ± 2.5	76.9
Drug incompatibility (4)	2.7 ± 1.9	67.5
Drug storage (20)	14.7 ± 3.5	73.5

### PIM

The proportion of PIMs prescribed to oldest old patients with polypharmacy was 27.1%. Based on the prescriptions of the 258 subjects, there were a total of 70 PIMs prescribed for 61 patients. The specific distribution is shown in Table 6.

**Table 6:** Distribution of PIMs

PIM	Drug	*n*	%
	Central nervous system drugs: estazolam, alprazolam	10	14.3
Drugs that should be avoided by elderly people	Gastrointestinal drugs: metoclopramide	6	8.6
	Anticholinergic drugs: diphenhydramine, chlorpheniramine	3	4.3
	Spironolactone > 25 mg/d	5	7.1
	Digoxin > 0.125 mg/d	4	5.7
	Amiodarone, propafenone	3	4.3
	Short-acting nifedipine	7	10.0
	Prazosin, terazosin	4	5.7
	Ibuprofen, indomethacin	5	7.1
	Liquid paraffin	5	7.1
Elderly-specific disease state-related PIMs	Aspirin for heart failure	3	4.3
	Benzodiazepines for dementia and cognitive impairment	2	2.9
	Metoclopramide for Parkinson’s disease	3	4.3
	Theophylline for insomnia	2	2.9
Drugs that should be used with caution by elderly people	Aspirin for primary prevention of cardiovascular events	8	11.4

### Compliance

In this study, only 32.6% of the oldest old patients with polypharmacy were compliant to their treatment regimen. Of the four items on the Morisky scale, compliance was the lowest (50.0%) for forgetting to take medicines and the highest (59.3%) for sometimes being careless about taking medicine.

### Factors influencing compliance

Ten variables (gender, age, education level, marital status, medical costs, disease diagnosis, medication classes, quantity of drugs, adverse drug reactions, and medication knowledge) were analyzed using univariate and multivariate logistic regression analyses to determine the factors associated with compliance. The univariate logistic regression analysis showed that gender (*P* < 0.001), age (*P* < 0.001), marital status (*P* = 0.001), medical expenses (*P* = 0.003), disease diagnosis (*P* < 0.001), medication classes (*P* = 0.001), number of drugs (*P* = 0.002), and medication knowledge (*P* < 0.001) were associated with compliance. These variables were entered in the multivariate logistic regression analysis, revealing that age and medication classes were independently negatively associated with compliance, and that medication knowledge was independently positively associated with compliance (Table 7).

**Table 7:** Multivariate logistic regression analysis

Variable	OR (95% CI)	*P*
Age	0.36 (0.21–0.63)	< 0.001
Medication types	0.85 (0.75–0.95)	0.004
Medication knowledge	1.06 (1.02–1.10)	0.004

## Discussion

### Characteristics of the subjects

In the present study, oldest old of ≥ 80 years of age were specifically selected because they represent a relatively novel and expanding population in China’s aging society [32]. The majority of the patients in our study lived with their spouses, which is consistent with Chinese national data and the social characteristics of oldest old living arrangements [32]. This suggests that most of the subjects did not lack for care and had some form of physical and psychological support. Regarding health care costs, 96.8% of the subjects received free medical care or had health insurance, suggesting that the patients’ economic burden was light and that few of them were concerned about their medical expenses. The average number of chronic diseases per patient observed in this study was generally consistent with previous studies in China [33, 34]. The most commonly used prescription drugs were gastrointestinal drugs. Classification statistics was conducted in the cardiovascular drugs as follow: calcium antagonists, beta blockers, thiazide drugs, angiotensin, antithrombotic drugs and statins. So the cardiovascular drugs were very commonly used in older adults if aggregated in this study..

### Medication knowledge and compliance

Previous studies showed that appropriate medication knowledge was present in 60–72% of people [35–37]. In the present study, the subjects specifically lacked knowledge about drug side effects, drug abuse, and medication dosage, as supported by previous studies worldwide [38, 39]. These findings may be related to the participants’ advanced age, as the regression analysis results showed that medication knowledge declined with increasing age, which is supported by previous studies in different populations worldwide [40–42]. Previous studies showed that medication knowledge is intimately associated with compliance [35, 37, 39, 43, 44], but since multiple factors are involved in compliance, medication knowledge is thought to play a relatively minor role [45]. In this study, the rate of medication compliance among the oldest old patients with polypharmacy was lower than in previous studies [14, 15, 18, 39, 41, 46, 47]. Patients may forget to take their drugs because of poor self-management, poor memory, or a limited emphasis on taking routine medication [48]. It is known that medication compliance among the older adults is affected by many factors, including patient-related factors, social support, medical expenses, and the doctor-patient relationship [14, 15, 18, 39, 41, 46–48]. Although certain social demographic characteristics (e.g. age and gender) and disease factors cannot be changed, several patient-related factors (e.g., medication knowledge level) can be intervened on, improving compliance. This study showed that age and medication classes were independently associated medication compliance, and that medication knowledge was an independent protective factor, as supported by previous studies [48–50], and could be more important to medication compliance than previously suggested [45]; nevertheless, the present study examined Chinese patients ≥ 80 years old, while Lam et al. [45] examined Australian patients ≥ 65 years of age.

The use of an excessive number of types of drugs can increase the risk of adverse drug reactions and the complexity of dosing (e.g., the method of administration and frequency of medication use). The numbers of drugs used in the present study were higher than those reported in previous studies [12, 51, 52]. Traditional Chinese medicine must be considered since it is generally not used outside China, is counted when evaluating polypharmacy, and can lead to adverse drug reactions [53]. In the present study, the proportion of elderly patients taking traditional Chinese medicine was high, highlighting the potentially high impact of traditional Chinese medicine on polypharmacy in Chinese populations. In addition, the proportion of oldest old patients using over-the-counter health care products in our study was high, which was likely related to the light economic burden of the subjects. The numbers of drugs may influence patients’ compliance to treatment. Indeed, patients may worry that excessive drug use could lead to adverse reactions, which may affect compliance, as supported by the fact that 40.3% of the subjects in our study had a history of adverse drug reactions, which was higher than that of previous studies [47, 54, 55]. Therefore, before drugs are prescribed, health care providers should inform their oldest old patients of the expected effects of treatment and possible side effects.

### PIM

The frequency of PIM in this study was 27%. A study from the Middle East showed a PIM frequency of 52.5% [56]. A study from Taiwan revealed PIM frequencies varying between 24 and 73% among different hospitals [57]. In Europe, the frequency of PIM is around 20% [58], while it ranges from 14 to 27% in the United States [59]. Therefore, the frequency of PIM reported in this study was comparable to that observed in other countries, and lower than in previous studies in China. Indeed, among elderly ≥ 65 years of age, Li et al. reported a PIM frequency of 52% or 72%, depending upon the definition used [60], while Zhang et al. [61] showed PIM rates of 45–54% among hospitalized patients. These discrepancies could be due to the study population (in this study, oldest old ≥ 80 years of age), the definition of PIM, and period covered (here, pre-admission). The benzodiazepines were commonly used of PIMs In this study. The oldest old who took the benzodiazepines were more likely to be cognitive and psychomotor impairment compared with young people. So No-drug Therapy is advised firstly for the oldest old with insomnia.

### Limits

The present study is not without limitations. The sample size was relatively small and from a single center. The selection of the patients will inevitably result in biases, affecting the generalizability of the results. Finally, no control group was included and the analyses were mostly descriptive, limited the internal and external validity of the results. Additional studies are still necessary to improve drug management in elderly patients.

## Conclusion

This study indicates that oldest old patients ≥ 80 years old and with polypharmacy in China showed a low medication knowledge, low medication compliance, and high frequency of PIMs. Several factors influencing medication compliance were identified, and the findings could provide a basis for the study of rational drug use in oldest old patients with polypharmacy.

## Additional files

Additional file 1: Medication knowledge questionnaire (non-validated English version). (DOCX 17 kb)

Additional file 2: Medication review chart (non-validated English version). (DOC 43 kb)

## Abbreviations

ADR: Adverse Reaction
CVI: Content Validity Index
HIS: Hospital Information System
PIM: Potentially Inappropriate Medication
TCM: Traditional Chinese Medication
