# Supplementary material for: Polypharmacy in the oldest old (≥80 years of age) patients in China: a cross-sectional study
Source: BMC Geriatr. 2018 Mar 2;18:64. doi: 10.1186/s12877-018-0754-y (PMC5834886; doi:10.1186/s12877-018-0754-y)
Supplement: Supplementary file 1 — Medication knowledge questionnaire (non-validated English version). (DOCX 17 kb) [file 12877_2018_754_MOESM1_ESM.docx]

**Medication knowledge questionnaire (non-validated English version)**

Answer the following questions according to your own understanding. Please check your answer in the corresponding box.

1. Drugs are divided into prescription and non-prescription drugs.

🞏Yes 🞏Uncertain 🞏No

1. Prescription drug is abbreviated as OTC.

🞏 Yes 🞏Uncertain 🞏No

1. Drugs have both therapeutic and toxic side-effects.

🞏 Yes 🞏Uncertain 🞏No

1. Traditional Chinese medicine or Chinese traditional patent medicine also has toxic side-effects.

🞏 Yes 🞏Uncertain 🞏No

1. Tonic medicine is good for the body, without harm.

🞏 Yes 🞏Uncertain 🞏No

1. The more the types of drugs, the better the treatment effect.

🞏 Yes 🞏Uncertain 🞏No

1. Drug dosage in the elderly is smaller than that in the adults.

🞏 Yes 🞏Uncertain 🞏No

1. The incidence of adverse reactions in the elderly is smaller than that in the general population.

🞏 Yes 🞏Uncertain 🞏No

1. For the people suffering from the same disease, the type and dosage of drug should be the same.

🞏 Yes 🞏Uncertain 🞏No

1. The more the types and dosage of drugs, the more prone to adverse drug reactions.

🞏 Yes 🞏Uncertain 🞏No

1. The drug can be discontinued if the condition is improved and there is no discomfort.

🞏 Yes 🞏Uncertain 🞏No

1. Medication can be adjusted without doctor’s permission.

🞏 Yes 🞏Uncertain 🞏No

1. Medication time has no effect on the efficacy of drug.

🞏 Yes 🞏Uncertain 🞏No

1. When the body is unwell, drug treatment is used first rather than diet, exercise or psychotherapy.

🞏 Yes 🞏Uncertain 🞏No

1. Indiscriminate use of certain drugs can cause drug resistance.

🞏 Yes 🞏Uncertain 🞏No

1. Antibiotics can be used for a long time or in large quantities.

🞏 Yes 🞏Uncertain 🞏No

1. Some drugs can be addictive for long-term use.

🞏 Yes 🞏Uncertain 🞏No

1. Once adverse reactions occur after medication, the drug should be discontinued in time .

🞏 Yes 🞏Uncertain 🞏No

1. I know how to judge the quality of drugs.

🞏 Yes 🞏Uncertain 🞏No

1. Drugs can be continued if they are moist, softened, caking, discolored or precipitated.

🞏 Yes 🞏Uncertain 🞏No

1. Self-purchased health care drugs can be taken at will.

🞏 Yes 🞏Uncertain 🞏No

1. Expired drugs can also be used.

🞏 Yes 🞏Uncertain 🞏No

1. Metamorphic drugs cannot be used.

🞏 Yes 🞏Uncertain 🞏No

1. You can take medicine with milk, tea, or drink.

🞏 Yes 🞏Uncertain 🞏No

1. Drugs should be placed in shade, avoid light.

🞏 Yes 🞏Uncertain 🞏No
