# Supplementary material for: Polypharmacy in the oldest old (≥80 years of age) patients in China: a cross-sectional study
Source: BMC Geriatr. 2018 Mar 2;18:64. doi: 10.1186/s12877-018-0754-y (PMC5834886; doi:10.1186/s12877-018-0754-y)
Supplement: Supplementary file 2 — Medication review chart (non-validated English version). (DOC 43 kb) [file 12877_2018_754_MOESM2_ESM.doc]

**medication review chart (non-validated English version)**

| Name： | | | | | | Date: / / | | |
| --- | --- | --- | --- | --- | --- | --- | --- | --- |
| ID： | | | | | |  |  |  |
| Drugs | Dosage | Number（pill/ capsule） | | | | Aim and remarks | Start date | Withdrawal date |
|  |  | morning | afternoon | evening | bedtime |  |  |  |
|  |  |  |  |  |  |  |  |  |
|  |  |  |  |  |  |  |  |  |
|  |  |  |  |  |  |  |  |  |
|  |  |  |  |  |  |  |  |  |
|  |  |  |  |  |  |  |  |  |
|  |  |  |  |  |  |  |  |  |
|  |  |  |  |  |  |  |  |  |
|  |  |  |  |  |  |  |  |  |
|  |  |  |  |  |  |  |  |  |
|  |  |  |  |  |  |  |  |  |
|  |  |  |  |  |  |  |  |  |
|  |  |  |  |  |  |  |  |  |
|  |  |  |  |  |  |  |  |  |
